# Novel *In Vitro* Screening System Based on Differential Scanning Fluorimetry to Search for Small Molecules against the Disassembly or Assembly of HIV-1 Capsid Protein

**DOI:** 10.3389/fmicb.2017.01413

**Published:** 2017-07-24

**Authors:** Yasuyuki Miyazaki, Naoya Doi, Takaaki Koma, Akio Adachi, Masako Nomaguchi

**Affiliations:** ^1^Department of Microbiology and Cell Biology, Tokyo Metropolitan Institute of Medical Science Tokyo, Japan; ^2^Department of Microbiology, Tokushima University Graduate School of Medical Science Tokushima, Japan; ^3^Department of Microbiology, Kansai Medical University Osaka, Japan

**Keywords:** HIV-1, Gag-CA, CA-polymerization, CA-stability, NaCl, ZnCl_2_

## Abstract

Varieties of *in vitro* systems have been used to study biochemical properties of human immunodeficiency virus Gag-capsid protein (HIV Gag-CA). Recently, we have comparatively characterized HIV-1 and HIV-2 Gag-CA proteins using such technology, and have demonstrated that the NaCl-initiated CA-polymerization *in vitro* and the stability of CA N-terminal domain as judged by differential scanning fluorimetry (DSF) are inversely correlated. In this study, we found that ZnCl_2_ works as a competent initiator of the *in vitro* HIV-1 CA-polymerization at much lower concentrations than those of NaCl frequently used for the polymerization initiation. We also showed by DSF assays that ZnCl_2_ highly destabilize HIV-1 CA. Furthermore, PF74, a well-known inducer of premature HIV-1 uncoating in infected cells, was demonstrated to unusually promote the HIV-1 CA-disassembly in the presence of ZnCl_2_ as revealed by DSF assays. Taken together, we conclude that the DSF method may be useful as an efficient monitoring system to screen anti-HIV-1 CA molecules.

## Introduction

Functional core structure consisting of numerous capsid (CA) proteins is a major component of viral particles and is essential for the replication of human immunodeficiency virus type 1 (HIV-1) (Freed and Martin, 2013; Campbell and Hope, 2015; Yamashita and Engelman, 2017). As well-documented, Gag-CA plays critical multiple roles at various steps in the HIV-1 life cycle. It needs to be underscored that the biological and/or biochemical analysis from different angles of this multi-functional viral protein is a prerequisite to understand the virology of HIV-1 with a complicated replication mode.

Disassembly and assembly processes of Gag-CA in cells are definitely vital for HIV-1, and can be analyzed by *in vitro* systems using the purified proteins produced in bacteria (Ehrlich et al., 1992; Li et al., 2000; Ganser-Pornillos et al., 2004; Barklis et al., 2009; Miyazaki et al., 2017). In our previous study, we have demonstrated the NaCl-dependent increase in HIV-1 Gag-CA polymerization/assembly and also in HIV-1 Gag-CA N-terminal domain (NTD) instability (Miyazaki et al., 2017). In the work, we employed the turbidity and fluorescence-based thermal shift assays to monitor Gag-CA assembly and Gag-CA NTD stability, respectively (Miyazaki et al., 2017). Although Gag-CA self-assembly could be induced by NaCl, a high concentration (1–2 M) was required to initiate the reaction, being a potential obstacle to smoothly perform various experiments. The thermal shift assay, i.e., the differential scanning fluorimetry (DSF), makes use of different fluorescence intensities from the target protein differentially bound with SYPRO orange (Invitrogen) by heat denaturation. This DSF method could be applicable to various studies, and notably, is suitable for large-scale handlings of the samples. In the present study, we found that ZnCl_2_ can effectively induce Gag-CA polymerization with much lower concentrations relative to NaCl. We also showed by DSF assays that a small molecule with a known anti-CA property indeed gives an effect on the CA-stability, consistent with the anti-viral activity. We propose here that the DSF system can be applicable for searching for anti-HIV-1 CA antivirals.

## ZnCl_2_ Promotes Polymerization of HIV-1 CA and Destabilizes its NTD

**FIGURE 1:**
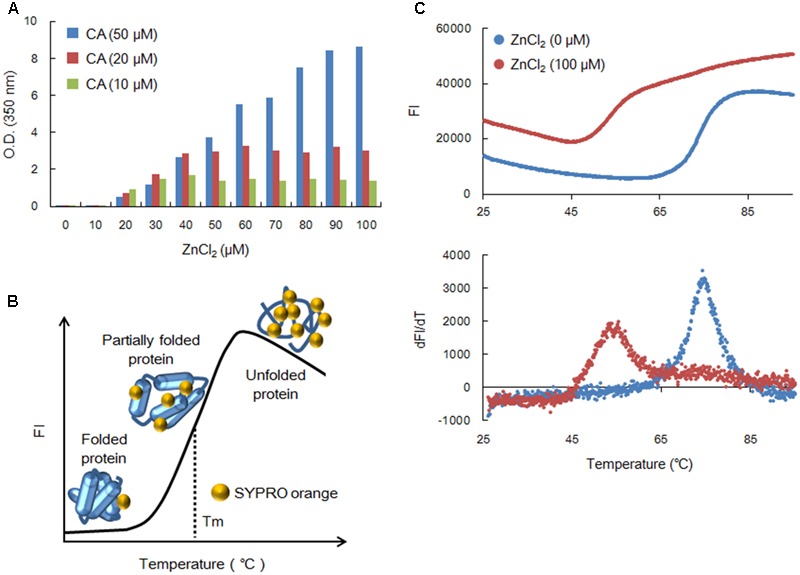
Assembly and thermal stability of NL4-3 Gag-CA. **(A)** Polymerization of Gag-CA. Length for polymerization reactions was 4 h as previously described (Miyazaki et al., 2017). Polymerized products were monitored by OD at 350 nm using a spectrophotometer (NanoDrop 1000, Thermo Fisher Scientific). **(B)** A schema for the DSF method. FI curve is illustrated by SYPRO orange FI values from various states of the test protein. **(C)** Thermal stability of NL4-3 Gag-CA NTD. This was determined as previously described (Miyazaki et al., 2017). SYPRO orange FI at different temperatures was monitored by 7500 real-time PCR system (Applied Biosystems), and melt curves were calculated by differences in FI at each temperature (dFI/dT). Peak temperatures in dFI/dT curves represent Tm. The experiments in **(A,C)** were repeated with similar results.

In this study, we always used histidine-tagged Gag-CA (Miyazaki et al., 2017), derived from an infectious clone of HIV-1 designated NL4-3 (Adachi et al., 1986), as the experimental material. We have previously analyzed the *in vitro* assembly property of HIV-1 CA mediated by high concentrations of NaCl (Miyazaki et al., 2017). In mature HIV-1 virions, four cleaved forms of Gag proteins, namely, matrix (MA), CA, nucleocapsid (NC), and p6, are present in close proximity (Freed and Martin, 2013). Because of the nature of these Gag mature products, we examined the effect of NC, which contains two zinc-binding motif (Freed and Martin, 2013), on the polymerization and thermal stability HIV-1 Gag-CA. During the study, we noticed that ZnCl_2_ could promote the HIV-1 Gag-CA assembly *in vitro*. We therefore asked whether the CA-polymerization is dependent on ZnCl_2_ concentrations. Polymerization process was monitored by measuring the optical density (OD) of the reaction at 350 nm using a spectrophotometer (NanoDrop 1000, Thermo Fisher Scientific). As shown in **Figure 1A**, although the reactions with 10 and 20 μM CA gave a plateau at relatively high concentrations of ZnCl_2_, the polymerization with 50 μM CA proceeded linearly with respect to the ZnCl_2_ concentrations up to 100 μM. This result is a sharp contrast to the previous data of the NaCl-initiated polymerization assays, in which at least 10^6^ μM NaCl was required to detect a significant level of polymerized products (Miyazaki et al., 2017). Although the molecular basis for this difference remains to be determined, it is conceivable that the polymerization could be efficiently triggered through enhanced complex formation of histidine-tagged CA by Zn^2+^. In any case, it is clear that ZnCl_2_ is a better reagent than NaCl to set up experiments here.

We then compared the thermal stability of NL4-3 CA NTD in the presence (100 μM) and absence of ZnCl_2_ by the DSF method using SYPRO orange as described before (Niesen et al., 2007; Fedorov et al., 2012; Miyazaki et al., 2017). SYPRO orange binds to hydrophobic patches of the test protein exposed by heat treatment (**Figure 1B**). As can be seen, the extent of heat denaturation, i.e., the thermal stability of the protein, is quantitatively estimated by the fluorescence intensity (FI) from SYPRO orange bound to the protein. **Figure 1C** shows the actual results of the assay. From the FI and peak temperatures in the melt curves that are calculated by the difference in FI at each temperature (dFI/dT), the Tm values (melting temperature) for CA-NTD at zero and 100 μM of ZnCl_2_ were found to be 74.1 and 54.8°C, respectively. Thus, consistent with the enhancement of CA-polymerization, ZnCl_2_ destabilized the CA-NTD very efficiently at much lower concentrations, again in contrast to the effective concentrations reported for NaCl (Miyazaki et al., 2017).

## System to Search for Anti-CA Molecules

**FIGURE 2:**
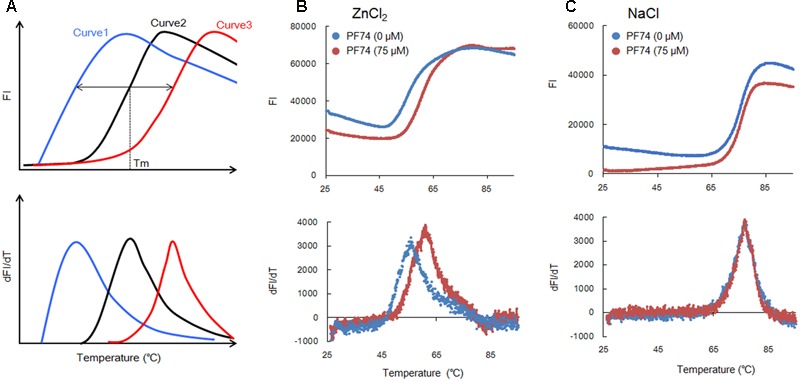
Thermal stability curves of NL4-3 Gag-CA NTD. **(A)** Schematic representation of the stability curves. Curves 1 and 3 show the results in the presence of molecules relatively destabilizing and stabilizing CA-NTD protein, respectively, compared to a control (curve 2, in the absence of any molecules interfering with the CA-assembly). This schema is depicted based upon the assumption that FI values are similarly minimal at lower temperatures and increase/decrease similarly at higher temperatures under various conditions. **(B,C)** Thermal stability of Gag-CA NTD in the absence and presence of PF74 (75 μM). The curves obtained for reactions with ZnCl_2_ (100 μM) or NaCl (100 μM) are shown in **(B,C)**, respectively, as indicated. SYPRO orange FI at different temperatures was monitored by 7500 real-time PCR system (Applied Biosystems), and melt curves were calculated by differences in FI at each temperature (dFI/dT). Peak temperatures in dFI/dT curves represent Tm. The results in **(B,C)** were reproduced in experiments using lower concentrations of PF74 (25 and 50 μM).

Together with the data here (**Figure 1C**) and our previous results (Miyazaki et al., 2017), we could propose a new system based on the DSF assay to screen or identify molecules that aberrantly destabilize or stabilize HIV-CA NTD, a rate-limiting viral factor for the CA-polymerization (Lingappa et al., 2014). DSF system can be used as a simple and rapid method to search for small molecule modulators of CA assembly. We routinely use a real-time PCR machine such as the 7500 system (Applied Biosystems) to perform DSF assays to assure that large numbers of test samples can be automatically handled in a short time. **Figure 2A** depicts a schema to explain the basis for the method (**Figure 1C**) (Miyazaki et al., 2017). In a control, i.e., in the absence of any molecules with anti-CA assembly, CA exhibits its own thermal stability as revealed by FI (curve 2) and dFI/dT. If some test molecules are present in the FI monitoring assay system that relatively destabilize (curve 1: promote assembly) or stabilize (curve 3: promote disassembly) the CA-NTD protein as compared to a negative control, decreased (curve 1) or increased (curve 3) Tm-shift would be expected. Because the FI curves simply reflect the SYPRO orange-binding status of the test proteins (**Figure 1B**), basal levels of the FI values would be variable somewhat under conditions used. However, these variations intrinsically have no effects on the Tm value itself.

To verify the above working hypothesis, we have selected PF74 that was recently reported to be an effective anti-HIV-CA small molecule, and assessed its activity against HIV-1 CA by the DSF assay. PF74 has been precisely analyzed by both *in vitro* and *in vivo* experiments for its anti-virus activity and the underlying molecular mechanism (Blair et al., 2010; Shi et al., 2011, 2015; Bhattacharya et al., 2014; Lad et al., 2015). PF74 binds to HIV-1 CA-NTD and promotes the assembly of the CA proteins *in vitro* (Blair et al., 2010; Bhattacharya et al., 2014; Lad et al., 2015), whereas it destabilizes assembled CA proteins (Shi et al., 2011). These seemingly paradoxical results can likely be explained by a notion that PF74 acts against HIV-1 replication through aberrantly destabilizing higher-order structures of CA proteins, i.e., the core-like structures, observed *in vitro* (Ehrlich et al., 1992; Li et al., 2000; Ganser-Pornillos et al., 2004; Barklis et al., 2009; Miyazaki et al., 2017). Indeed, the capsid structure in cells and virions was found to be affected by PF74 (Blair et al., 2010; Shi et al., 2011; Bhattacharya et al., 2014). Consistent with the above thought, *in vivo* analyses at a cellular level have shown that PF74 does not affect virus production but makes nascent virions virtually non-infectious (Blair et al., 2010; Shi et al., 2011). Furthermore, it has been revealed that PF74 targets an early virus replication phase, probably the viral uncoating step (Blair et al., 2010; Shi et al., 2011). Collectively, it is rational to conclude that PF74 exerts its anti-HIV-1 effect at the early viral replication stage to inhibit the reverse transcription of viral genomic RNA in infected cells. Thus, PF74 promotes premature virus uncoating, exhibiting an anti-HIV-1 activity similar to that of a cellular anti-viral restriction factor designated TRIM5α (Malim and Bieniasz, 2012; Nakayama and Shioda, 2015). Of note here, in our *in vitro* DSF system, both the assembly and disassembly of HIV-1 CA-NTD proteins could occur. The data obtained by this system would represent the resultant sum of both processes.

As shown in **Figure 2B**, PF74 (75 μM) shifted the Tm as seen for curve 3 in **Figure 2A**. Based on Tm values with and without PF74, 60.9 and 55.4°C, respectively, we concluded that it enhances the CA-disassembly. This observation was compatible with the findings summarized above (Blair et al., 2010; Shi et al., 2011; Bhattacharya et al., 2014). In a concurrent experiment, we also determined whether PF74 affects the stability of HIV-1 CA-NTD in the presence of NaCl (100 μM). As readily seen in **Figure 2C**, no appreciable difference in the Tm values with and without PF74 was detected (76.2 and 76.6°C, respectively). Therefore, the result in **Figure 2B** may be significant, since the Tm-shift was observed only when the CA-polymerization could occur. While ZnCl_2_ (100 μM) efficiently induced the CA-assembly (**Figure 1A**), NaCl (100 μM) did not initiate the process at all (Miyazaki et al., 2017). In total, we provided experimental evidence to show that our DSF system consisting of HIV-1 CA-NTD and fluorescence-based thermal shift assay is useful for screening anti-HIV-1 molecules targeting its CA.

## Concluding Remarks

In pilot experiments, we have successfully used a high-throughput screening method, i.e., the DSF assay, to explore for anti-HIV-1 CA small molecules (**Figures 1, 2**). In the assay system, HIV-1 CA-NTD protein (50 μM) is prepared in 50 mM Tris–HCl (pH 8.0), 100 μM ZnCl_2_, and 1 mM 2-mercaptethanol containing SYPRO orange, and analyzed through a temperature gradient (25–95°C) using a real-time PCR machine (Niesen et al., 2007; Fedorov et al., 2012; Miyazaki et al., 2017). The turbidity assay of CA assembly can also be developed into a high-throughput screening method, but the DSF system has the clear advantage over it. The DSF method is more sensitive than the turbidity assay, and gives more quantitative and reproducible data to select molecules with unusually CA-destabilizing or -stabilizing activity. Our DSF system is simple, and all reagents including the CA-NTD protein are readily available. Although this method is promising, it is absolutely necessary to test large numbers of molecules against HIV-1 CA, and to find certain candidates deserve evaluation of their inhibitory effects on the HIV-1 replication in cells.

## Author Contributions

YM, AA, and MN designed the research project. YM performed the experiments. YM, ND, TK, AA, and MN discussed the results. YM, AA, and MN wrote the manuscript. All authors approved its submission.

## Conflict of Interest Statement

The authors declare that the research was conducted in the absence of any commercial or financial relationships that could be construed as a potential conflict of interest.
